# Pros and Cons of Early and Late Skin Grafting in Children with Burns—Evaluation of Common Concepts

**DOI:** 10.3390/ebj3010015

**Published:** 2022-02-22

**Authors:** Islam Abdelrahman, Ingrid Steinvall, Folke Sjöberg, Mohamed A. Ellabban, Johann Zdolsek, Moustafa Elmasry

**Affiliations:** 1 Department of Hand Surgery, Plastic Surgery and Burns and Department of Biomedical and Clinical Sciences, Linköping University, 58183 Linköping, Sweden; ingrid.steinvall@regionostergotland.se (I.S.); folke.sjoberg@liu.se (F.S.); johann.zdolsek@regionostergotland.se (J.Z.); moustafa.elmasry@liu.se (M.E.); 2 Department of Anaesthesiology and Intensive Care, Linköping University, 58183 Linköping, Sweden; 3 Plastic and Reconstructive Surgery Unit, Department of Surgery, Suez Canal University, Ismailia 41522, Egypt; ellabban2005@yahoo.com

**Keywords:** burns, children, healing time, burn surgery, skin graft

## Abstract

Background: There is no consensus regarding the timing of surgery in children with smaller burn size, specifically in deep dermal burns. Delayed surgery has risks in terms of infection and delayed wound healing. Early surgery also risks the removal of potentially viable tissue. Our aim was to investigate the effect of the timing of surgical intervention on the size of the area operated on and the time to wound healing. Methods: A retrospective analysis for all children (<18 years) with burn size <20% body surface area (BSA%) during 2009–2020 who were operated on with a split-thickness skin graft. The patients were grouped by the timing of the first skin graft operation: early = operated on within 14 days of injury; delayed = operated on more than two weeks after injury. Results: A total of 84 patients were included in the study, 43 who had an early operation and 41 who had a delayed operation. There were no differences between the groups regarding burn size, or whether the burns were superficial or deep. The mean duration of healing time was seven days longer in the group with delayed operation (*p* = 0.001). The area operated on was somewhat larger (not significantly so) in the group who had early operation. Nine children had two skin graft operations, eight in the early group and one in the delayed group (*p* = 0.03). Conclusion: The patients who were operated on early had the advantage of a shorter healing time, but there was a higher rate of complementary operations and a tendency towards a larger burn excision.

## 1. Introduction

The assessment of the wound depth in burns is an essential step for the choice of treatment strategy. Partial-thickness injuries can be classified into two groups according to depth (i.e., superficial dermal and deep dermal burns). Superficial dermal burns typically heal spontaneously within two weeks. Deep dermal and full thickness burns usually require surgery. The clinical assessment of superficial and full-thickness burns is usually easy, but the assessment of deep dermal burns can be challenging [1]. The gold standard management of deep burns in adults, either full thickness or deep dermal, is early excision and skin grafting of the wound [2,3,4].

There is, however, no consensus on the timing of surgery in children with smaller burn size, particularly in deep dermal burns, as many surgeons prefer to wait until the demarcation between superficial and deep burns has been established, which usually occurs within two weeks after injury [5,6]. Delayed surgery entails a higher frequency of infection as well as delayed wound healing, which in adults has been shown to be associated with worse scarring [4]. A recent study has shown that non-excisional mechanical debridement of burns among children can promote epithelialisation and decrease the odds of requiring a skin graft [7]. Early surgery can also risk the removal of potentially viable tissue. Excising an unnecessarily large burn area will consequently result in the need for a larger skin graft [5]. There is a clinical dilemma in finding the optimal timing for the excision of deep dermal burns in children. An important requirement for early excision is to identify the deep wound in a mixed-depth burn as early as possible after injury.

To the best of our knowledge, there are few studies investigating the optimal timing of surgery in children with deep dermal burns. Partial-thickness burns in children have been studied from other perspectives, such as exploring healing time using different types of dressings [8] and some studies have shown a higher risk for hypertrophic scarring among children with longer healing time [9].

We wanted to highlight the pros and cons of early and late skin grafting in children with burns. Our aim was therefore to investigate the effect of the timing of surgical intervention (i.e., early versus late) on the size of the area operated on, time to wound healing, and the requirement of extra operations in children with burns.

## 2. Methods

**Figure 1:**
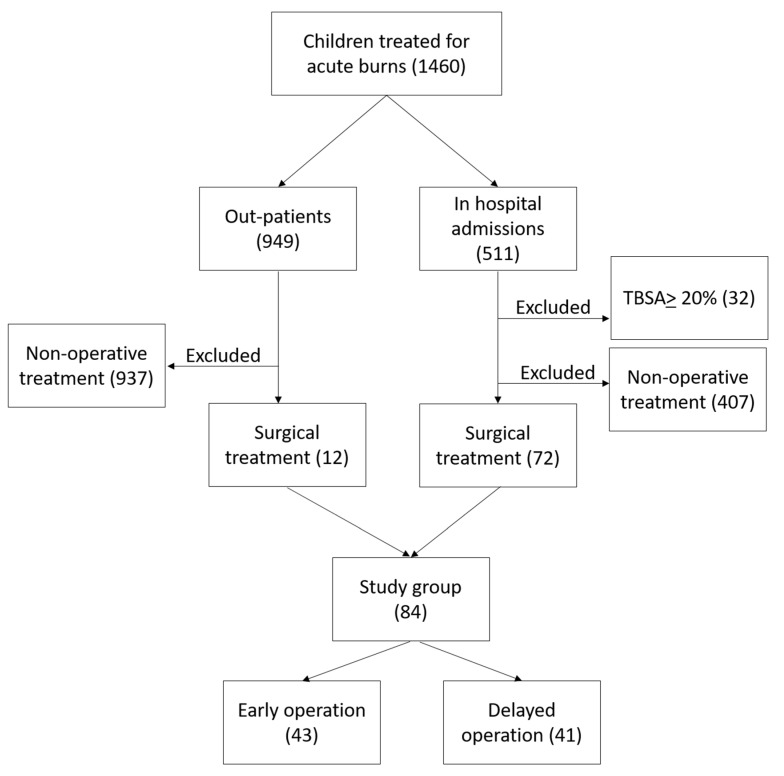
Patient selection: Flowchart for children managed at Linköping Burn Centre during the period 2009–2020.

A retrospective analysis was made of the records of all children less than 18 years old with burns, managed at Linköping Burn Centre during the period 2009–2020, who were operated on with split-thickness skin grafts at any time during the acute management phase. We included patients younger than 18 years with burns between 1 and 19.9% total body surface area percentage (TBSA%). We excluded patients with TBSA% burned of 20% and more, as well as patients who completed their treatment in other hospitals. All children who did not need a surgical intervention by mean of skin graft were excluded from the study. The retrieval of data was performed using our local burns database and hospital medical records. See patient selection in Figure 1.

The variables used were total burn size; superficial, deep dermal and full-thickness burns; age; type of burn; timing between injury and first operation with skin grafting; wound healing time (defined as the period between injury to the date of the visit in which the wound showed epithelisation of at least 95% to both grafted and non-grafted areas); the area operated on; and preoperative and postoperative treatment with antibiotics. The patients were grouped into two cohorts by the timing of the first skin graft operation: early = operated on within the first two weeks of injury; delayed = operated on more than two weeks after injury.

The extent and depth of the burns were examined at admission by a burn surgeon who took into account the appearance of the wound, capillary refill, and the sensory functions of the injured areas. The assessment was recorded on a Lund and Browder chart. 

All superficial burns were treated conservatively for 14 days, in the hope of spontaneous healing. Deeper burns were always evaluated by a specialist/consultant in burn surgery during the initial period to decide which areas were to be excised immediately and which to be treated conservatively (non-excisional mechanical debridement and wound dressing) until demarcation of the deep burns. Reassessment of the wound depth was performed regularly with the dressing change with antimicrobial foam dressing, which usually occurred 2–3 times a week. All dressings were performed under sedation in the out-patient clinic. The strategy was to not excise viable tissue, to minimise the total treatment period.

The criteria for antibiotic administration are rising evels of C-reactive protein concentration (CRP), fever more than 38 degrees C and/or visible signs of wound infection. We do not have the strategy of giving prophylactic antibiotics [10].

### Statistics

Descriptive data are presented as mean (SD) or No. (%). The Mann–Whitney U and chi squared (or Fisher’s exact) tests were used for analysis of two groups and the Kruskal–Wallis ANOVA for analysis of more than two groups. Probabilities of less than 0.05 were accepted as significant.

## 3. Results

A total of 84 patients were included in the study, 43 with early operation and 41 with delayed operation. There were no significant differences between the groups regarding burn size, superficial or deep burns, burn type, age, and sex, although there was a tendency towards younger patients in the group with delayed operation. Antibiotic administration before and after operation was observed in almost half of the children in both groups and there was no difference between the groups (Table 1). There was no difference in timing of the first operation between patient with scalds compared to the other burn types (*p* = 0.41). Supplemental Table S1 shows the same variables as Table 1 but with a different grouping in which early operation was divided into two groups (first and second week).

The mean duration of healing time was seven days longer in the group with delayed operation, while the period from operation to healing did not differ significantly. Supplemental Table S1 shows that this pattern of prolonged healing time remained significant even when the patients were subdivided into weekly groups. Healing time in the delayed operation group was longer in the subgroup of scalds (*p* = 0.005, *n* = 25 and 29) and flame burns (*p* = 0.04, *n* = 8 and 6) but not among contact burns (*p* = 0.22, *n* = 10 and 6). Healing time was almost double among the patients with flame burns who were operated late (Table 2).

**Figure 2:**
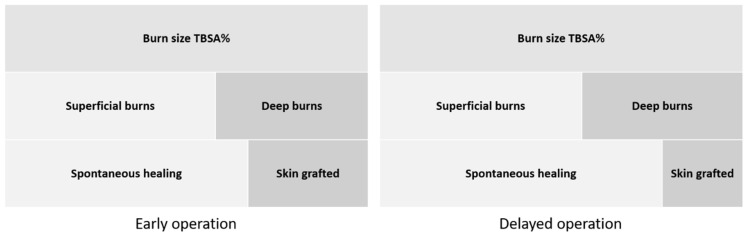
Schematic description of the (mean) size of the area operated on in relation to the size of superficial and deep burns in both groups. TBSA% = percentage total body surface area.

The area operated on was somewhat larger (not significantly so) in the early operation group, while the size of deep burns was similar in both groups. Figure 2 shows a schematic description of the relation between the area of skin grafted and burn size.

Nine children had two operations, eight in the early group and one in the delayed group (Table 1). The eight patients who had two operations in the early group had a mean of 14 days longer healing time than the other 35 patients who were operated on early. Otherwise, there were no significant differences in burn size, depth, age, or days from injury to first operation (Table 3).

In the early group, the most common reason for reoperation was incomplete graft take, followed by infection, and deepening of the superficial wound area. The only patient with a second skin graft operation in the delayed group had partial graft failure due to haematoma (Table 4).

## 4. Discussion

We found that healing time was shorter among the patients operated on within the first two weeks after injury compared with those operated on after two weeks. We also noticed a higher frequency of reoperations in the early group, and a tendency towards a larger excised area (although the latter was not significant). This is, to our knowledge, the first study to investigate the effect of the timing of the skin-grafting operations among children with small burns. The burn size in the current study was small and most of the children were treated on an out-patients basis after the skin graft operation [11].

There is a tendency in clinical practice to balance the urgency of early excision with the known background of the better healing capacity of children [12]. Experimental studies have shown the benefits of early excision to modulate the inflammatory process [13].

The treatment strategy with early excision and skin grafting of the wound has shown better results in reducing the rates of mortality and infection [14], as well as better hand mobility [15]. These results were not specific for a paediatric population. There are publications that have shown better results in mortality and morbidity among children with severe burns when operated on early [16,17,18,19]. However, the drawbacks of an early operation shown in our study were the high frequency of reoperation and tendency towards larger excisions. This could be explained partly by difficulties in determining which areas were to be excised. The ongoing research to determine the depth of the burn as early as possible could improve the accuracy of the size of the excised area [20,21]. Another explanation is the progression of burn depth, a multifactorial process caused by variables such as a prolonged inflammatory response, local ischaemia, hypercoagulability, wound infection, and deficit in nutrition [22,23,24].

We found a lower rate of second skin-graft operations in the delayed group, which is considered an advantage of following this strategy. Two weeks after injury, most wounds are well demarcated, which reduces the risk of excising vital tissues and missing areas that need surgery [5]. It was shown that the children in the late group were younger, although not significantly so. This reflects the conservative approach taken with younger children in the hopes that the wounds would heal by secondary intention without a need for surgery.

### 4.1. Healing Time

Our results of longer healing time by delayed operation were similar to those of previous studies. Lonie et al. reported 25 children with scalds who were operated on with skin grafts a mean of 16 days after injury. Healing time with operation on day seven was 15–21 days, healing time with operation on day 14 was 22–30 days, and healing time was more than 30 days with operation on day 18 [25]. A relatively large variation in healing time has also previously been reported. Jewell et al. showed that time to complete wound healing ranged from 2 to 75 days among all admissions for burns who had skin grafts; one-quarter were children and two-thirds had a burn size ≤10% TBSA [26]. Gravante et al., on the other hand, reported a smaller variation in a standardised study group of otherwise healthy adults with small burns that required excision and skin grafts: mean (SD) healing time of 12 (2) days [27].

Compared to the group who were operated on later, we found a shorter healing time among the subgroup of patients with flame burns who were operated on early. These results can be used to improve future treatment for this group. We did not find a significant difference regarding time between skin-graft operation and wound healing between the groups, therefore we think that the modifiable factor to decrease the total healing time is to shorten the period between injury and skin-graft operation.

Increased knowledge of the optimal timing of skin-graft surgery would benefit both healthcare providers and patients. The benefits of this include a reduction in the risk of complications such as wound infection and sepsis. It also helps to reduce the number of visits for dressing changes, which are usually performed under sedation and require more resources. This would also benefit the families who seek care outside the county in terms of less parental leave and better psychological well-being for the children. A shorter wound-healing time would, in many patients, most likely result in the healed wound having a better quality of scar. Additionally, laser treatment can be used to improve the final outcome of the scar [28]. Regardless of the knowledge gained from studies such as ours, the treatment plan for each patient should be tailored individually according to preferences and social circumstance.

### 4.2. Limitations

The most important limitation of this study was the fact that it was a retrospective, single-centre study based on a restricted number of observations. The one-centre approach makes it difficult to generalise the result, but has the advantages of a more homogeneous population and less variation in treatments, which can strengthen the value of the results. Furthermore, we did not undergo big changes in the local practice including dressing materials and frequency during this period. Another limitation was that the study was not designed to inspect the reasons behind the timing of operations. It is difficult to interpret the reasoning for these decisions from the medical records alone. Finally, we did not have long-term follow up of scar quality in the present study.

## 5. Conclusions

The patients who were operated on early had the advantage of a shorter healing time; however, there was a higher rate of complementary operations and a tendency towards a larger burn excision.

## Figures and Tables

**Table 1:** Details of the patients.

Variables	All	Early Operation	Delayed Operation	*p* Value
No. of patients	84	43 (51%)	41 (49%)	
Age, years	5.6 (5.4)	6.4 (5.5)	4.7 (5.2)	0.14
Sex, male	52 (62%)	25 (58%)	27 (66%)	0.47
Burn size, TBSA%:	6.1 (5.4)	6.1 (5.8)	6.0 (5.1)	0.68
Superficial dermal burn, BSA%	3.5 (4.5)	3.6 (5.2)	3.4 (3.8)	0.44
Deep dermal and full-thickness burn, BSA%	2.5 (3.9)	2.5 (3.7)	2.5 (4.1)	0.67
Operated and skin-grafted area, BSA%	1.7 (2.2)	2.0 (2.7)	1.3 (1.5)	0.24
Burn type				0.46
Scald	54 (64%)	25 (58%)	29 (71%)	
Contact burn	16 (19%)	10 (23%)	6 (15%)	
Flame burn	14 (17%)	8 (19%)	6 (15%)	
Patients with two operations	9 (11%)	8 (19%)	1 (2%)	0.03
Healing time, days from injury	30.7 (12.2)	26.9 (11.8)	34.7 (11.5)	0.001
Days from injury to operation	13.4 (6.7)	8.3 (3.5)	18.7 (4.9)	<0.001
Days from operation to healing	17.4 (10.5)	18.6 (11.1)	16.0 (9.7)	0.20
Antibiotic administration (before operation)	56%	53%	59%	0.56
Antibiotic administration (after operation)	51%	59%	43%	0.17

Data are presented as mean (SD) or *n* (%). The *p* values are calculated on the two groups (early and late operation). Mann–Whitney U and chi squared or Fisher’s exact tests as appropriate. TBSA% = percentage total body surface area. BSA% = percentage body surface area.

**Table 2:** Healing time by burn type.

Burn Type	Early Operation	Delayed Operation	*p* Value
Scald	25.0 (10.8)	31.2 (8.7)	0.005
Flame burn	26.6 (15.4)	48.8 (15.2)	0.04
Contact burn	32.0 (10.7)	37.8 (8.7)	0.22

Data are presented as mean (SD). Mann–Whitney U test.

**Table 3:** Details of the early group by number of operations.

Variables	One Operation	Two Operations	*p* Value
No. of patients	35	8	
Days from injury to first operation	8.4 (3.4)	7.6 (4.0)	0.59
Age, years	6.0 (5.3)	8.0 (6.6)	0.47
Burn size, TBSA%:	5.7 (5.7)	7.9 (6.3)	0.29
Superficial dermal burn, BSA%	3.5 (5.3)	4.4 (4.8)	0.25
Deep dermal and full thickness burn, BSA%	2.2 (3.0)	3.6 (6.2)	0.82
Operated and skin-grafted area, BSA%	1.4 (1.6)	4.5 (4.6)	0.07
Healing time, days from injury	24.3 (10.3)	38.4 (11.5)	0.004
Days between the two operations		14.1 (8.2)	

Data are presented as mean (SD). Mann–Whitney U. TBSA% = percentage total body surface area. BSA% = percentage body surface area.

**Table 4:** Reasons for the second skin graft operation.

Group	Operated Area *	Infection	Incomplete Graft Take	Haematoma	Deepening of a Superficial Area
Early	0.8	x			
Early	4				x
Early	1	x	x		
Early	6		x		
Early	13		x		
Early	9		x		x
Early	2				x
Early	0.3	x			
Delayed	1.5			x	

* Operated and skin-grafted area (BSA%) in the first operation.

## Data Availability

The dataset used and/or analysed during the current study are available from the corresponding author on reasonable request.

## Supplementary Materials

The following supporting information can be downloaded at: https://www.mdpi.com/article/10.3390/ebj3010015/s1, Supplemental Table S1: Details of the patients grouped by timing of first operation.
