# Supplementary material for: Pros and Cons of Early and Late Skin Grafting in Children with Burns—Evaluation of Common Concepts
Source: Eur Burn J. 2022 Feb 22;3(1):180–7. doi: 10.3390/ebj3010015 (PMC11575360; doi:10.3390/ebj3010015)
Supplement: Supplementary file 1 [file ebj-03-00015-s001.zip › ebj-1585008-supplementary.pdf]

european burn journal
MDPI

Table S1. Details of the patients grouped by timing of first operation.

Variables	ALL	Week 1	Week 2	Week 3 and Later	ANOVA/Chi Square	1 & 2	1 & 3	2 & 3
No. of patients	84	14 (17%)	29 (35%)	41 (49%)				
Age, years	5.6 (5.4)	11.1 (5.5)	4.1 (3.9)	4.7 (5.2)	0.002	<0.001	0.001	0.98
Sex, male	52 (62%)	7 (50%)	18 (62%)	27 (66%)	0.57			
Burn size, TBSA%	6.1 (5.4)	4.5 (4.7)	6.9 (6.2)	6.0 (5.1)	0.37			
Superficial dermal burn, BSA%	3.5 (4.5)	2.1 (3.5)	4.4 (5.7)	3.4 (3.8)	0.37			
Deep dermal and full thickness burn, BSA%	2.5 (3.9)	2.3 (2.7)	2.5 (4.2)	2.5 (4.1)	0.68			
Operated and skin grafted area, BSA%	1.7 (2.2)	2.0 (2.6)	2.0 (2.8)	1.3 (1.5)	0.48			
Burn type					0.43			
Scald	54 (64%)	6 (43%)	19 (66%)	29 (71%)				
Contact burn	16 (19%)	4 (29%)	6 (21%)	6 (15%)				
Flame burn	14 (17%)	4 (29%)	4 (14%)	6 (15%)				
Patients with two operations	9 (11%)	4 (29%)	4 (14%)	1 (2%)	0.02	0.40	0.01	0.15
Healing time, days from injury	30.7 (12.2)	22.3 (14.1)	29.1 (10.0)	34.7 (11.5)	0.001	0.02	<0.001	0.03
Days from injury to operation	13.4 (6.7)	4.1 (2.0)	10.3 (1.9)	18.7 (4.9)	<0.001	<0.001	<0.001	<0.001
Days from operation to healing	17.4 (10.5)	18.1 (14.0)	18.9 (9.7)	16.0 (9.7)	0.33			

Antibiotic administration (before operation)	56%	33%	61%	59%	0.24
Antibiotic administration (after operation)	51%	50%	63%	43%	0.30

Mean (SD) or n (%). Kruskal-Wallis ANOVA, post hoc Mann-Whitney U test. Chi squared test (post hoc Fisher exact test). Post hoc analysis was done pairwise, group 1 = first operation day 0-7, group 2 = first operation day 7-13, group 3 = first operation day ≥14. TBSA%, percentage total body surface area. BSA%, percentage body surface area.
